# Gender‐specific association between body mass index and all‐cause mortality in patients with atrial fibrillation

**DOI:** 10.1002/clc.23371

**Published:** 2020-04-30

**Authors:** Si‐qi Lyu, Yan‐min Yang, Jun Zhu, Juan Wang, Shuang Wu, Han Zhang, Xing‐hui Shao, Jia‐meng Ren

**Affiliations:** ^1^ Emergency and Critical Care Center, State Key Laboratory of Cardiovascular Disease, Fuwai Hospital, National Center for Cardiovascular Diseases, Chinese Academy of Medical Sciences and Peking Union Medical College Beijing People's Republic of China

**Keywords:** atrial fibrillation, body mass index, gender, all‐cause mortality

## Abstract

**Background:**

Elevated body mass index (BMI) is related with reduced mortality in various cardiovascular diseases.

**Hypothesis:**

Gender‐specific association between BMI and mortality exists in atrial fibrillation (AF).

**Methods:**

In this multicenter observational study with a mean follow‐up of 1 year, a total of 1991 AF patients were enrolled and divided into two groups based on the gender. The primary endpoint was all‐cause mortality while the secondary endpoints were defined as cardiovascular mortality, stroke, and major adverse events during 1‐year follow‐up. Cox regression was performed to identify the association between BMI and clinical outcomes according to gender.

**Results:**

Female patients with AF tended to be older (*P* = .027) and thinner (*P* < .001) than male patients with AF. They were more likely to have heart failure, hyperthyroidism, and valvular AF (all *P* < .05), but less likely to have coronary artery disease and prior myocardial infarction (all *P* < .01). Multivariate analysis revealed that overweight (HR(95%CI): 0.55(0.41‐0.75), *P* < .001) and obese patients (HR(95%CI): 0.56(0.34‐0.94), *P* = .028) were associated with significant lower all‐cause mortality compared with normal weight patients for the entire cohort. Similar association between elevated BMI and reduced all‐cause mortality were only identified in female patients with AF (overweight vs normal weight: HR(95%CI): 0.43(0.27‐0.70); obesity vs normal weight: HR(95%CI): 0.46(0.22‐0.97)), but not in male patients with AF.

**Conclusion:**

This study indicates that overweight and obesity were related with improved survival in patients with AF. The association between elevated BMI and reduced mortality was dependent on gender, which was only significant in female patients, rather than male patients.

## 1 INTRODUCTION

As a global health issue, the prevalence of overweight and obesity has increased rapidly over the years due to lifestyle changes.^1^ Elevated body mass index (BMI) is a well‐established risk factor for various cardiovascular diseases such as hypertension, diabetes mellitus, heart failure, and coronary artery disease.^2^ Atrial fibrillation (AF) is one of the most common arrhythmia and is related with notably higher incidence of substantial complications such as stroke and heart failure.^3^, ^4^ In patients with AF, overweight and obesity are prevalent and are verified to increase the risk of AF occurrence and recurrence.^5^, ^6^

It is well established that overweight and obesity are associated with increased mortality in the general population.^7^ On the contrary, a plenty of studies have demonstrated that higher BMI might be associated with lower mortality in the setting of various cardiovascular diseases including hypertension,^8^ diabetes,^9^ coronary artery disease,^10^ heart failure,^11^, ^12^ stroke or transient ischemic attack (TIA).^13^ In patients with AF, the counterintuitive association between elevated BMI and reduced mortality has also been detected, which is referred to as “obesity paradox.”^14^

Significant disparities exist in the presentation and mechanism of obesity between male and female patients.^15^ Previous studies have suggested that the relation between obesity and mortality might be dependent on gender, not only in general population,^16^ but also in patients with heart failure,^11^, ^12^ coronary artery disease,^17^ and chronic kidney disease.^18^ In patients with AF, the clinical characteristics and prognosis are also quite different between male and female patients.^19^, ^20^ However, little is known about whether gender has an impact on the association between obesity and mortality in AF patients. Therefore, a multicenter observational study conducted in Chinese AF patients was utilized to explore this issue.

## 2 METHODS

### 2.1 Study population

This multicenter observational study was designed to consecutively enroll patients presenting to emergency department with a diagnosis of AF. Twenty representative hospitals around China (including rural and urban, academic and community, general and specialized, public and private hospitals) have participated in this study. The diagnosis of AF was confirmed by reviewing clinical records, electrocardiographic evidence, and electronic databases according to International Classification of Disease, 9th Revision, Clinical Modification Diagnostic Code 427.31 or 427.32. The study was approved by the ethics committee of each center and obeyed the Declaration of Helsinki. All patients have provided written consent to participate in this study.

### 2.2 Baseline

Baseline data about demographics information, medical histories, admission vital signs, and treatments were collected by interviewing the patients, consulting their treating physicians and reviewing their medical records. BMI was recorded on admission and calculated by dividing weight in kilograms by the square of height in meters. AF was classified to paroxysmal, persistent, and permanent according to guidelines.^3^ Comorbidities including hypertension, diabetes mellitus, heart failure, coronary artery disease, prior myocardial infarction, left ventricular hypertrophy, congenital heart disease, valvular AF, stroke/TIA, chronic obstructive pulmonary disease (COPD), dementia, hyperthyroidism, prior major bleeding and tobacco use, were obtained based on the medical records. Valvular AF was defined as AF related to moderate or severe mitral stenosis or a history of mechanical valve replacement. 3 Risk of stroke was assessed by CHA2DS2‐VASc score (congestive heart failure, one point; hypertension, one point; age ≥75 years, two points; age 65‐74 years, one point; diabetes mellitus, one point; stroke, two points; vascular disease, one point; female gender, one point). Treatments including rate and rhythm control agents, anticoagulants, and antiplatelet agents, were also collected.

### 2.3 Follow‐up and outcomes

Follow‐up was carried out by trained research personnel via telephone interview, outpatient visit, or medical records procurement, with a mean duration of 1 year. The primary endpoint was all‐cause mortality, including cardiovascular and non‐cardiovascular mortality. The secondary endpoints were defined as cardiovascular mortality, stroke, and major adverse events (MAEs) during 1‐year follow‐up. All outcomes were adjudicated by an independent committee blinded to the patients according to standardized definitions. Deaths and its causes were determined by medical records obtained and reports of the participants' relatives or physicians. Cardiovascular deaths included deaths due to heart failure, myocardial infarction, sudden/arrhythmic death, stroke, pulmonary embolus, peripheral embolus, aortic dissection, or other cardiovascular disorders. MAEs referred to composite endpoint events of all‐cause mortality, stroke, non‐central nervous system (CNS) embolism, and major bleeding. Stroke was defined as focal neurological deficits lasting more than 24 hours and confirmed by imaging. Non‐CNS embolism was defined as a vascular occlusion due to embolism confirmed by imaging or surgery. Major bleeding was defined as life‐threatening bleeding, and/or symptomatic bleeding in a critical area or organ, such as intra‐cranial, or pericardial, or intra muscular with compartment syndrome, and/or bleeding causing a fall in hemoglobin level of 20 g/L (1.24 mmol/L) or more, or leading to transfusion of two or more units of whole blood or red cells.

### 2.4 Statistical analysis

Continuous variables were presented as medians with interquartile ranges and compared by Mann‐Whitney *U* test for the data were not normally distributed. Categorical variables were presented as frequencies and percentages and compared by Chi‐square test. BMI was evaluated both as a continuous variable and as a categorical variable. According to Chinese criteria of weight for adults, patients were divided into four categories: underweight (BMI < 18.5 kg/m^2^), normal weight (BMI 18.5‐23.9 kg/m^2^), overweight (BMI 24‐27.9 kg/m^2^), and obesity (BMI ≥ 28 kg/m^2^).^21^ Kaplan‐Meier curves for 1‐year all‐cause mortality and cardiovascular mortality were constructed and log‐rank tests were used to compare survival discrepancies across BMI strata in patients with AF. Univariate and multivariate Cox proportional hazard regression were performed for all‐cause mortality, cardiovascular mortality, stroke, and MAEs, while hazard ratio (HR) and 95% confidence interval (CI) were calculated. Variables with a *P* value <.10 in the univariate analysis and factors relevant with outcomes were entered into the multivariate Cox models for adjustment. All statistical tests were two‐sided, and a *P* value <.05 was considered significant. SPSS 25.0 (IBM Corporation, New York) was used for statistical analysis.

## 3 RESULTS

Between September 2008 and April 2011, 2016 patients presenting to emergency department with documented AF were recruited. Among them, 25 patients were excluded due to incomplete data. Consequently, a total of 1991 patients with a median age of 71 years and 1093 females (54.9%) were included in the final analysis and divided into the male group and the female group.

The baseline characteristics and treatments of patients with AF are shown in Table 1. Compared to male patients with AF, female patients with AF tended to be older (*P* = .027) and thinner (*P* < .001). They were more likely to have heart failure, hyperthyroidism and valvular AF (all *P* < .05), and less likely to have coronary artery disease and prior myocardial infarction (all *P* < .01). As to medications, there was no significant difference according to gender, except that female patients had a higher rate of digoxin use (*P* = .034). When CHA2DS2‐VASc score was calculated, 623(69.4%) male patients and 987(90.3%) female patients had a CHA2DS2‐VASc score ≥ 2 (*P* < 0.001). However, among the patients with prior diagnosis of AF and anticoagulant indications, only about 20% of them have received anticoagulant therapy at enrollment.

**TABLE 1:** Baseline characteristics of male and female patients with AF

Characteristic	Male (n = 898)	Female (n = 1093)	All (n = 1991)	*P* value
*Demographics*, n (*%*)				
Age (y)	70 (58‐77)	72 (61‐79)	71 (60‐78)	.027
BMI (kg/m^2^)	23.8 (21.6‐26.0)	23.0 (21.0‐25.2)	23.4 (21.3‐25.7)	<.001
*BMI categories*, n (*%*)				
Underweight (BMI < 18.5)	51 (5.7%)	108 (9.9%)	159 (8.0%)	<.001
Normal weight (BMI 18.5–23.9)	422 (47.0%)	560 (51.2%)	982 (49.3%)	
Overweight (BMI 24–27.9)	322 (35.9%)	316 (28.9%)	638 (32.0%)	
Obesity (BMI ≥ 28)	103 (11.5%)	109 (10.0%)	212 (10.6%)	
SBP (mm Hg)	130 (119‐147)	130 (116‐145.5)	130 (117‐146)	.690
DBP (mm Hg)	80 (70‐90)	80 (70‐90)	80 (70‐90)	.184
Ventricular rate (bpm)	96 (80‐120)	98 (80‐120)	97 (80‐120)	.643
Prior diagnosis of AF	719 (80.1%)	889 (81.3%)	1608 (80.8%)	.475
AF as a main diagnosis	373 (41.5%)	446 (40.8%)	819 (41.1%)	.741
*Type of AF*, n (*%*)				
Paroxysmal	282 (31.4%)	326 (29.8%)	608 (30.5%)	.673
Persistent	204 (22.7%)	245 (22.4%)	449 (22.6%)	
Permanent	412 (45.9%)	522 (47.8%)	934 (46.9%)	
*Medical histories*, n (*%*)				
Hypertension	492 (54.8%)	618 (56.5%)	1110 (55.8%)	.433
Diabetes mellitus	133 (14.8%)	176 (16.1%)	309(15.5%)	.428
Heart failure	310 (34.5%)	434 (39.7%)	744 (37.4%)	.017
Coronary artery disease	408 (45.4%)	427 (39.1%)	835 (41.9%)	.004
Prior myocardial infarction	86 (9.6%)	61 (5.6%)	147 (7.4%)	.001
Left ventricular hypertrophy	145 (16.1%)	177 (16.2%)	322 (16.2%)	.977
Congenital heart disease	22 (2.4%)	21 (1.9%)	43 (2.2%)	.419
Valvular AF	67 (7.5%)	186 (17.0%)	253 (12.7%)	<.001
Stroke/TIA	181 (20.2%)	193 (17.7%)	374 (18.8%)	.156
Dementia	21 (2.3%)	23 (2.1%)	44 (2.2%)	.724
Hyperthyroidism	18 (2.0%)	48 (4.4%)	66 (3.3%)	.003
COPD	114 (12.7%)	114 (10.4%)	228 (11.5%)	.114
Tobacco use	370 (41.2%)	55 (5.0%)	425 (21.3%)	<.001
CHA2DS2‐VASc score ≥2	623 (69.4%)	987 (90.3%)	1610 (80.9%)	<.001
Prior major bleeding	27 (3.0%)	21 (1.9%)	48 (2.4%)	.116
*Medication status*, n (*%*)				
β blocker	397 (44.2%)	477 (43.6%)	874 (43.9%)	.799
ACEI/ARB	349 (38.9%)	410 (37.5%)	759 (38.1%)	.536
Calcium channel blocker	198 (22.0%)	263 (24.1%)	461 (23.2%)	.289
Digoxin	253 (28.2%)	356 (32.6%)	609 (30.6%)	.034
Amiodarone	87 (9.7%)	104 (9.5%)	191 (9.6%)	.896
Propafenone	25 (2.8%)	28 (2.6%)	53 (2.7%)	.759
Anticoagulant	185 (20.6%)	259 (23.7%)	444 (22.3%)	.099
Aspirin	506 (56.3%)	586 (53.6%)	1092 (54.8%)	.223
Statin	226 (25.2%)	250 (22.9%)	476 (23.9%)	.232
Diuretic	331 (36.9%)	437 (40.0%)	768 (38.6%)	.154

Abbreviations: ACEI, angiotensin‐converting enzyme inhibitors; AF, atrial fibrillation; ARB, angiotensin receptor blockers; BMI, body mass index; COPD, chronic obstructive pulmonary disease; DBP, diastolic blood pressure; SBP, systolic blood pressure; TIA transient ischemic attack.

The 1‐year outcomes of patients with AF are shown in Table 2. During 1‐year follow‐up, a total of 277 all‐cause deaths occurred, including 168 cardiovascular deaths, in which 151 patients have suffered from stroke, while 29 patients have experienced major bleeding. Compared to male patients with AF, female patients with AF had comparable all‐cause mortality (13.4% vs 14.5%, *P* = .510), cardiovascular mortality (9.0% vs 7.8%, *P* = .350) and MAEs (21.2% vs 20.9%, *P* = .874), but a significantly higher incidence of stroke (8.7% vs 6.2%, *P* = .039).

**TABLE 2:** 1‐Year outcomes in male and female patients with AF according to BMI categories

	Male						Female					
	Underweight (n = 51)	Normal weight (n = 422)	Overweight (n = 322)	Obesity (n = 103)	All (n = 898)	*P* value	Underweight (n = 108)	Normal weight (n = 560)	Overweight (n = 316)	Obesity (n = 109)	All (n = 1093)	*P* value
All‐cause mortality	12 (23.5%)	74 (17.5%)	35 (10.9%)	9 (8.7%)	130 (14.5%)	.005	24 (22.2%)	93 (16.6%)	22 (7.0%)	8 (7.3%)	147 (13.4%)	<0.001
Cardiovascular mortality	7 (13.7%)	35 (8.3%)	21 (6.5%)	7 (6.8%)	70 (7.8%)	.372	19 (17.6%)	59 (10.5%)	13 (4.1%)	7 (6.4%)	98 (9.0%)	<0.001
Stroke	3 (5.9%)	27 (6.4%)	21 (6.5%)	5 (4.9%)	56 (6.2%)	.933	10 (9.3%)	46 (8.2%)	30 (9.5%)	9 (8.3%)	95 (8.7%)	.922
MAEs	12 (23.5%)	100 (23.7%)	62 (19.3%)	14 (13.6%)	188 (20.9%)	.109	30 (27.8%)	131 (23.4%)	50 (15.8%)	21 (19.3%)	232 (21.2%)	.018
Non‐CNS embolism	1 (2.0%)	1 (0.2%)	8 (2.5%)	2 (1.9%)	12 (1.3%)	.032	0 (0.0%)	5 (0.9%)	2 (0.6%)	5 (4.6%)	12 (1.1%)	.017
Major bleeding	2 (3.9%)	5 (1.2%)	9 (2.8%)	2 (1.9%)	18 (2.0%)	.340	2 (1.9%)	7 (1.3%)	1 (0.3%)	1 (0.9%)	11 (1.0%)	.391

Abbreviations: AF, atrial fibrillation; BMI, body mass index; CNS, central nervous system; DM, diabetes mellitus; MAEs, major adverse events.

**FIGURE 1:**
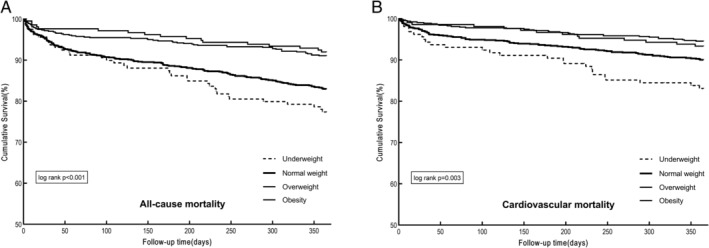
1‐Year Kaplan–Meier survival curves of patients with atrial fibrillation (AF) according to BMI categories: A, all‐cause mortality and B, cardiovascular mortality

Figure 1 displays the 1‐year Kaplan‐Meier survival curves across different BMI strata. In the entire cohort, all‐cause mortality (*P* < .001) and cardiovascular mortality (*P* = 0.003) decreased significantly with the increase of BMI.

**FIGURE 2:**
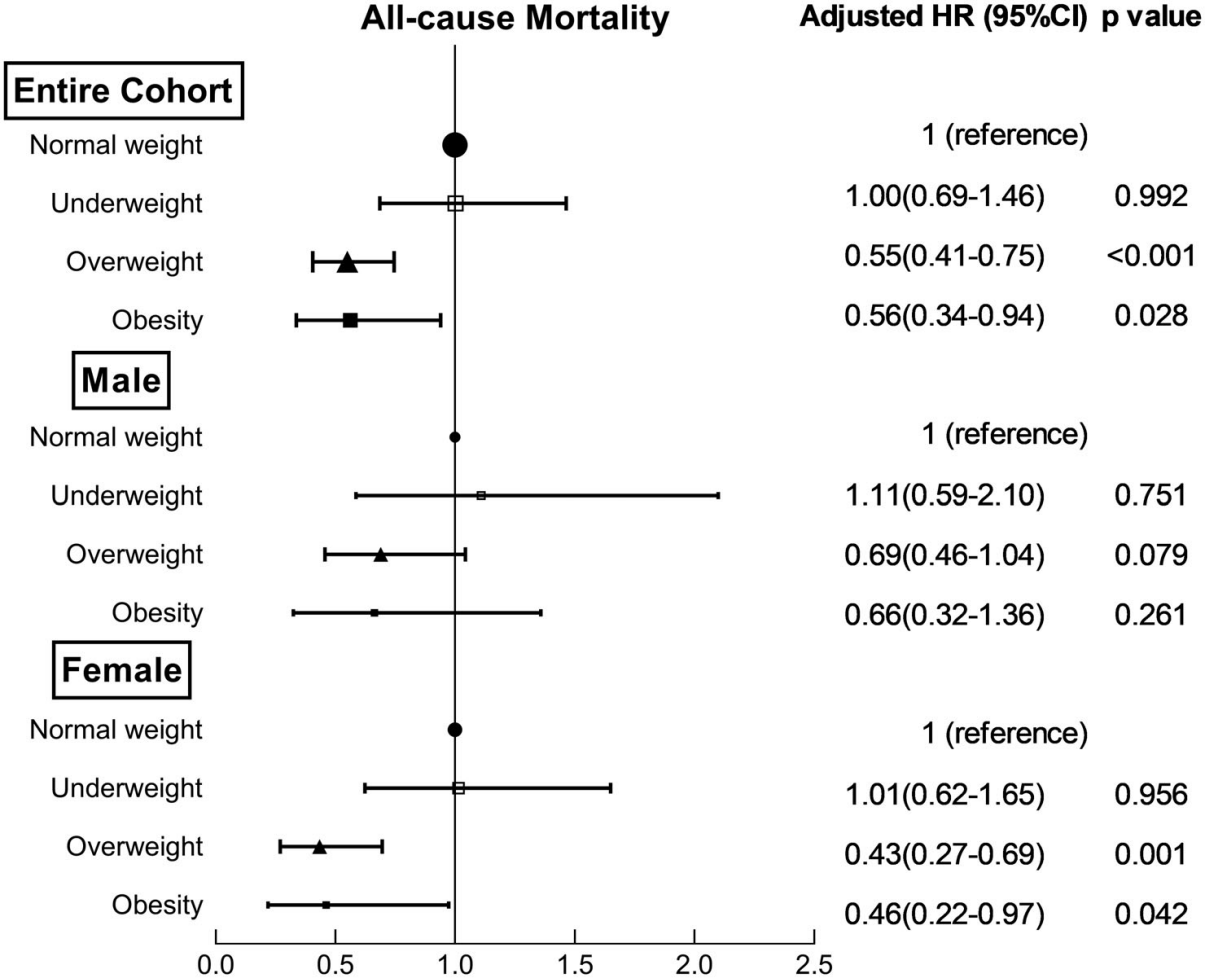
Multivariate adjusted hazard ratios of all‐cause mortality categorized by sex and body mass index (BMI)

Whether entered into the univariate Cox regression as a continuous variable or as a categorical variable, increased BMI was recognized to be a protective predictor for 1‐year all‐cause mortality, cardiovascular mortality, and MAEs (Table 3). In the multivariate regression, age, gender, systolic blood pressure, diastolic blood pressure, AF as a main diagnosis, type of AF, hypertension, diabetes mellitus, heart failure, coronary artery disease, prior myocardial infarction, valvular AF, stroke/TIA, dementia, COPD, tobacco use, β blocker, angiotensin‐converting enzyme inhibitor/angiotensin receptor blocker, calcium channel blocker, digoxin, anticoagulant, statin, and diuretic were entered with backward stepwise method. After adjustment for relevant covariates, the association between higher BMI and better outcomes still existed. Compared with normal weight patients, overweight (HR(95%CI): 0.55(0.41‐0.75), *P* < .001) and obese patients (HR(95%CI): 0.56(0.34‐0.94), *P* = .028) were associated with significantly reduced all‐cause mortality for the entire cohort. Moreover, the overweight group had notably lower risk of cardiovascular mortality (HR(95%CI): 0.54(0.37‐0.81), *P* = .003) and MAEs (HR(95%CI): 0.79(0.63‐0.99), *P* = .042). In female patients with AF, overweight (HR(95%CI): 0.43(0.27‐0.70), *P* = .001) and obesity (HR(95%CI): 0.46(0.22‐0.97), *P* = .042) were still remarkable predictors for decreased all‐cause mortality. However, all‐cause mortality was comparable across BMI strata in male patients with AF (Figure 2).

**TABLE 3:** Univariate and multivariate Cox analysis of 1‐year outcomes entering BMI as a continuous and categorical variable, respectively

	All‐cause mortality		Cardiovascular mortality		Stroke		MAEs	
	HR (95% CI)	*P* value	HR (95% CI)	*P* value	HR (95% CI)	*P* value	HR (95% CI)	*P* value
*Univariate Cox regression*								
BMI (continuous), per 1 kg/m^2^	0.90 (0.87‐0.93)	<.001	0.88 (0.85‐0.92)	<.001	0.99 (0.95‐1.04)	.786	0.94 (0.92‐0.97)	<.001
*BMI* (*categorical*)								
Normal weight (BMI 18.5–23.9)	1 (reference)		1 (reference)		1 (reference)		1 (reference)	
Underweight (BMI < 18.5)	1.36 (0.95‐1.95)	.094	1.75 (1.13‐2.70)	.012	1.12 (0.62‐2.02)	.710	1.14 (0.82‐1.58)	.434
Overweight (BMI 24‐27.9)	0.50 (0.37‐0.68)	<.001	0.53 (0.36‐0.79)	.002	1.02 (0.72‐1.46)	.900	0.72 (0.57‐0.90)	.004
Obesity (BMI ≥ 28)	0.45 (0.27‐0.73)	.001	0.65 (0.37‐1.14)	.133	0.82 (0.47‐1.46)	.508	0.66 (0.46‐0.94)	.021
*Age‐adjusted HR* (*95%CI*)								
BMI (continuous), per 1 kg/m^2^	0.91 (0.88‐0.94)	<.001	0.89 (0.85‐0.93)	<.001	1.01 (0.96‐1.05)	.832	0.96 (0.93‐0.98)	.001
*BMI* (*categorical*)								
Normal weight (BMI 18.5–23.9)	1 (reference)		1 (reference)		1 (reference)		1 (reference)	
Underweight (BMI < 18.5)	1.25 (0.87‐1.80)	.222	1.69 (1.09‐2.60)	.019	1.09 (0.61‐1.97)	.772	1.09 (0.78‐1.52)	.607
Overweight (BMI 24–27.9)	0.55 (0.41–0.75)	<.001	0.57 (0.38‐0.84)	.004	1.09 (0.76‐1.56)	.634	0.78 (0.62‐0.97)	.029
Obesity (BMI ≥ 28)	0.53 (0.32‐0.88)	.013	0.74(0.42‐1.29)	.287	0.94(0.53‐1.66)	.818	0.77(0.539‐1.10)	.151
*Multivariate‐adjusted HR* (*95%CI*)								
BMI (continuous), per 1 kg/m^2^ ^a^	0.93 (0.90‐0.96)	<.001	0.92 (0.88‐0.96)	<.001	1.00 (0.95‐1.05)	.903	0.97 (0.94‐1.00)	.036
*BMI* (*categorical*)^a^								
Normal weight (BMI 18.5–23.9)	1 (reference)		1 (reference)		1 (reference)		1 (reference)	
Underweight (BMI < 18.5)	1.00 (0.69‐1.46)	.992	1.23 (0.78‐1.94)	.382	1.17 (0.63‐2.16)	.625	0.91 (0.65‐1.28)	.583
Overweight (BMI 24–27.9)	0.55 (0.41–0.75)	<.001	0.54 (0.37–0.81)	.003	1.10 (0.76‐1.58)	.617	0.79 (0.63–0.99)	.042
Obesity (BMI ≥ 28)	0.56 (0.34–0.94)	.028	0.71 (0.40‐1.28)	.255	0.92 (0.51‐1.65)	.773	0.81 (0.57‐1.18)	.272

Abbreviations: AF, atrial fibrillation; BMI, body mass index; CI, confidence interval; HR, hazard ratio; MAEs, major adverse events; TIA, transient ischemic attack.

a Adjusted for age, gender, systolic blood pressure, diastolic blood pressure, AF as a main diagnosis, type of AF, hypertension, diabetes mellitus, heart failure, coronary artery disease, prior myocardial infarction, valvular AF, stroke/TIA, dementia, chronic obstructive pulmonary disease, tobacco use, β blocker, angiotensin‐converting enzyme inhibitor/angiotensin receptor blocker, calcium channel blocker, digoxin, anticoagulant, statin, and diuretic.

## 4 DISCUSSION

In the present study, there were differences between male and female patients with AF in some aspect of clinical characteristics, comorbidities, medications, and prognosis. In the entire cohort, overweight and obesity were associated with significant lower all‐cause mortality, which was in‐line with the so‐called “obesity paradox.” However, when divided by gender, the association between elevated BMI and reduced all‐cause mortality was only significant in female patients, rather than in male patients.

Significant differences exist in the clinical characteristics and prognosis between male and female patients with AF.^19^ In this cohort, female patients with AF tended to have a lower rate of coronary artery disease and a higher rate of heart failure, hyperthyroidism, and valvular AF. During 1‐year follow‐up, they were more likely to suffer from stroke, which was consistent with previous reports.^19^

Obesity and overweight are well‐established risk factors for cardiovascular diseases, AF included.^1^, ^2^ Although elevated BMI was associated with increased mortality in general population,^16^ plentiful studies have demonstrated a converse relation between BMI and all‐cause mortality in the setting of numerous cardiovascular diseases.^8^, ^9^, ^10^, ^11^, ^12^, ^13^ As to patients with AF, the relation between BMI and mortality was controversial in previous studies.^14^, ^22^ In our study, AF patients in overweight and obesity groups had significantly lower risk of mortality than patients in normal weight group, which was in accordance with the obesity paradox.

The exact mechanisms of obesity paradox in AF have not been fully elucidated yet and might be multifactorial. One explanation is that obesity is a well‐established risk factor for heavier symptom burdens^6^ and more cardiovascular comorbidities in patients with AF.^14^, ^22^ These could lead to earlier diagnosis and better management. As a result, cardioprotective medications are adopted more frequently and aggressively in obese patients.^14^, ^22^ In addition, there might exist a “healthy survivor effect.” The prevalence of AF increases significantly with age.^3^, ^4^ Severe obese patients might die before developing AF due to comorbidities, leaving the rest obese AF patients with relatively favorable prognosis. In view of the above reasons, confounding effects should not be ignored in obesity paradox.^14^, ^22^ However, after adjustment for potential confounders, the relation between BMI and mortality still existed in our study. Another explanation might be that obese patients have better metabolic reserves to cope with increased metabolic stress in the setting of various diseases.^23^ AF is a kind of chronic disease with elevated energy and protein consumption, which is associated with worse prognosis.^3^ Adipose tissues could serve as energy reserves and play a positive role in delaying malnutrition and energy wastage caused by illnesses.^23^, ^24^ Meanwhile, elevated BMI could also be accompanied by higher muscle mass, which is beneficial for better survival.^24^, ^25^ On the other hand, adipose tissue could produce a variety of adipokines, which have a positive effect on myocardial metabolism.^26^ In addition, obese patients have a relatively lower level of atrial natriuretic peptide,^27^ a predictor for the prognosis of AF patients.^28^ This might be another potential explanation for obesity paradox in AF patients.

Several studies have demonstrated that there exist sex‐related differences in the phenomenon of obesity paradox. Vest et al found that overweight was associated with significant survival benefit only in female patients with heart failure,^11^ while another study of 3145 patients with heart failure revealed that obesity paradox only existed in female patients as well.^12^ Similar sexual dimorphism has also been discovered in patients with other diseases, such as coronary artery disease^17^ and chronic kidney disease.^18^ As to AF, analysis of 2540 patients in the EORP‐AF Registry demonstrated that all‐cause mortality increased with elevation of BMI in female patients (normal weight vs overweight vs obese: 9.3% vs 5.3% vs 4.3%, *P* = .023), but not in male patients (*P* = .748). However, multivariate regressions in male and female subgroup have not been performed in that study.^29^ Our study undertook multivariate Cox analysis in male and female patients, respectively, and indicated that overweight and obesity were associated with reduced mortality only in female patients with AF.

There might be several explanations for the sexual dimorphism in obesity paradox. First, previous studies suggested that female patients were associated with more severe AF symptom and worse quality of life, which might result in earlier diagnosis and better treatment.^19^, ^20^ On the other hand, female patients with AF in the present study had a significantly higher rate of valvular AF, which was a chronic consumptive disease and might benefit more from metabolic reserves of excessive adipose tissues.^3^ These confounding factors might partly account for the sex‐specific association between BMI and mortality. However, the result remained the same after adjustment for these potential confounding factors in the present study. Second, there exist sex‐related differences in the clinical characteristics of obese patients. BMI, a parameter combining both fat and lean body mass, is used to quantify overweight and obesity.^30^ Gender differences in body composition should not be ignored. Women usually have a higher percentage of fat mass than men with an equivalent BMI.^15^, ^30^ Since high percentage of fat mass is supposed to be protective for favorable survival due to better metabolic reserves, the relation between obesity and reduced mortality in female patients with chronic diseases is reasonable. On the other hand, the condition of fat distribution could not be reflected by BMI, either.^30^ Excessive fat stored in visceral fat deposits is more common in men, while in women, it is usually distributed in peripheral subcutaneous tissue.^15^, ^31^ Excessive visceral fat could increase the risk of developing metabolic syndrome and cardiovascular diseases, whereas femoral‐gluteal fat might be beneficial as a “sink” for lipid.^32^ Finally, greater myocardial fatty acid metabolism and lower myocardial glucose utilization have been observed in obese women, which might be partly attributed to the effect of estrogen.^33^ Estrogen can increase the activity of lipoprotein lipase and fatty acid oxidation enzyme, thus improving the myocardial fatty acid metabolism.^34^, ^35^ On the other hand, estrogen could reduce glucose oxidation, gluconeogenesis and glycogenolysis in other organs and decrease glucose transporter 4 translocation to the cell surface, thereby inhibiting glucose utilization.^35^, ^36^ Similar changes have also been detected in postmenopausal women with chronic estrogen replacement.^37^ In all, female myocardium might be more dependent on fatty acids metabolism for energy utilization than male myocardium. This could be one potential explanation for the survival advantages in female patients with obesity. In addition, excessive adipose tissue could increase the circulating levels of estrogen in obese women and exert further beneficial impacts on their prognosis.^38^, ^39^

The study had several limitations need to be pointed out. First, as one of the most common parameter of obesity, BMI has defects in reflecting the percentage and distribution of adipose tissues. However, our study lacked data of determinants for central obesity, such as waist circumference and waist‐to‐hip ratio, which have been confirmed to be better predictors of clinical outcomes.^40^ Second, only baseline BMI was available in the present study. The relation between BMI changes and outcomes was unknown due to lack of serial BMI during follow‐up. Third, some potentially relative factors, such as socioeconomic situation, cardiorespiratory fitness, nutritional status, unintentional and intentional weight loss, have not been collected and adjusted in this study. These residual confounders might have influenced the accuracy of our results. Finally, this is a post hoc observational study with inherent defects. The gender‐specific relation between BMI and mortality could only be interpreted as associative but not causal. Therefore, large prospective multicenter studies with rational design are needed to confirm our results.

## 5 CONCLUSION

Our study has demonstrated that overweight and obesity were related with decreased mortality in patients with AF. The association between elevated BMI and reduced mortality was dependent on gender. The phenomenon of obesity paradox could only be detected in female patients with AF. The exact mechanisms of the gender‐specific association between BMI and mortality have not been fully elucidated and require further investigation.

## CONFLICT OF INTEREST

The authors declare that they have no competing interests.

## AUTHOR CONTRIBUTIONS

Si‐qi Lyu: collected the data, performed the statistical analysis, drafted, and wrote the manuscript. Yan‐min Yang and Jun Zhu: designed and revised the manuscript. Shuang Wu, Juan Wang, Han Zhang, Xing‐hui Shao, and Jia‐meng Ren: collected the data. All the authors read and approved the final manuscript.
